# Early Postoperative Magnetic Resonance Imaging for Transsphenoidal Pituitary Surgery: A Systemic Literature Review and the Proposed Imaging Algorithm

**DOI:** 10.7759/cureus.77597

**Published:** 2025-01-17

**Authors:** Anurag Modak, Evan Gross, Andrew Yang, Maunil Mullick, Raja N Jani, Brian J Williams

**Affiliations:** 1 Neurosurgery, Rutgers New Jersey Medical School, Newark, USA; 2 Neurological Surgery, University of Alabama at Birmingham School of Medicine, Birmingham, USA; 3 Neurological Surgery, Weill Cornell Medicine, New York, USA; 4 Neurosurgery, University of Louisville School of Medicine, Louisville , USA; 5 Neurosurgery, University of Louisville School of Medicine, Louisville, USA

**Keywords:** endoscopic transsphenoidal pituitary surgery, magnetic resonance imaging and pituitary adenoma, pituitary adenoma management, postoperative outcomes, transsphenoidal neurosurgery

## Abstract

Current neurosurgical consensus guidelines recommend that the first radiologic study to evaluate the extent of pituitary tumor resection be performed 3-4 months after surgery, defined as late postoperative (LPO) magnetic resonance imaging (MRI), and include fat-suppressed T1 and T2 sequences. The current guidelines supporting LPO MRI stem from older studies which claim that imaging <3 months postoperatively, defined as early postoperative (EPO), cannot be reliably interpreted due to acute postoperative changes. Several new technical and technological innovations that emerged since the promulgation of these guidelines may allow neurosurgeons to evaluate the extent of pituitary adenoma resection within a shorter timeframe, with higher resolution, and with greater certainty of the surrounding anatomy. We therefore sought to develop an evidence-based imaging algorithm, with regard to timing, magnetic field strength, and choice of views, for postoperative MRI following transsphenoidal pituitary adenoma resection by performing a systematic review of the available literature.

## Introduction and background

Magnetic resonance imaging (MRI) is the standard tool for neuroradiologic evaluation of pituitary tumors. Although transsphenoidal resection has remained the most common treatment for craniopharyngiomas and non-prolactin-secreting adenomas for decades, the complex anatomy of the sellar and parasellar regions often precludes gross total resection (GTR) [1,2]. Reports suggest that GTR is achieved in only 50-80% of cases, highlighting the importance of postoperative MRI for evaluating the extent of residual tumor [3,4]. Early detection of residual tumors following pituitary adenoma resection may enable reoperation before the development of wound scarring and tumor adhesions that can complicate later surgical revisions [5].

Current neurosurgical consensus guidelines recommend that the first radiologic study to evaluate the extent of pituitary resection be performed 3-4 months after surgery, defined as late postoperative (LPO) imaging, and include fat-suppressed T1 and T2 sequences [6]. These guidelines also state that there is insufficient evidence to make recommendations for the frequency or duration of postoperative surveillance, but they recommend that patients undergoing GTR be followed less frequently than those undergoing subtotal resection [6]. The current guidelines supporting LPO MRI stem from older studies which claim that imaging <3 months postoperatively cannot be reliably interpreted due to acute postoperative changes such as cavity enlargement, hemorrhagic accumulation, and fat packing [1,7,8].

Several new techniques and technologies that may address the limitations noted by these prior studies have emerged. These innovations include the timing of MRIs, the magnetic field strength of the MRIs, and the views generated by the MRI. Examples include intraoperative MRIs, 3T MRIs, and dynamic sequences with contrast, among numerous others. Taken together, these new developments may allow neurosurgeons to evaluate the extent of pituitary adenoma resection within a shorter timeframe, with higher resolution, and with greater certainty of the surrounding anatomy.

Consequently, the literature addressing postoperative imaging after pituitary resection remains controversial. There is a growing body of evidence supporting the efficacy of early postoperative (EPO) MRI taken <3 months after pituitary resection, oftentimes as early as 48-72 hours postoperatively, and utilizing one or more of the aforementioned recently innovated techniques and technologies [9,10]. If reliably interpreted, EPO MRI allows for immediate intervention prior to the development of surgical site adhesions and scarring [11,12]. The primary objective of the current study was to develop an evidence-based imaging algorithm, with regard to timing, magnetic field strength, and choice of views, for EPO MRI following transsphenoidal pituitary adenoma resection by performing a systematic review of EPO MRI literature.

## Review

Methods

A systematic review was performed according to the Preferred Reporting Items for Systematic Reviews and Meta-Analyses (PRISMA) guidelines to determine if the currently used pituitary MRI sequences can accurately identify abnormalities in the EPO setting and to develop an evidence-based imaging algorithm. We determined that performing a systematic review was most appropriate given the specific nature of our research and our aim of proposing a novel pituitary imaging algorithm based on the existing literature.

A keyword search was conducted on PubMed, Scopus, Ovid, and the Cochrane Library. Articles were included if the title and/or abstract mentioned specific imaging views, timing of postoperative MRI, and strength of magnetic fields used as well as if the article was a cohort study, cross-sectional study, clinical trial, systematic review, or meta-analyses. Articles that did not meet these criteria or articles without English translation were excluded [13-15].

After reviewing the literature for imaging algorithms, the specific imaging views, timing of postoperative MRI, and strength of magnetic fields used were identified. For each identified setting of these variables, the quality of the evidence was assigned a classification based on the five-level system reported by Rothoerl et al. (2013) and Yarascavitch et al. (2012) in their systematic reviews of the quality of evidence in neurosurgical publications [16,17]. This classification system is ultimately derived from the guidelines published by the Oxford Center for Evidence-Based Medicine [18].

Results

**Figure 1:**
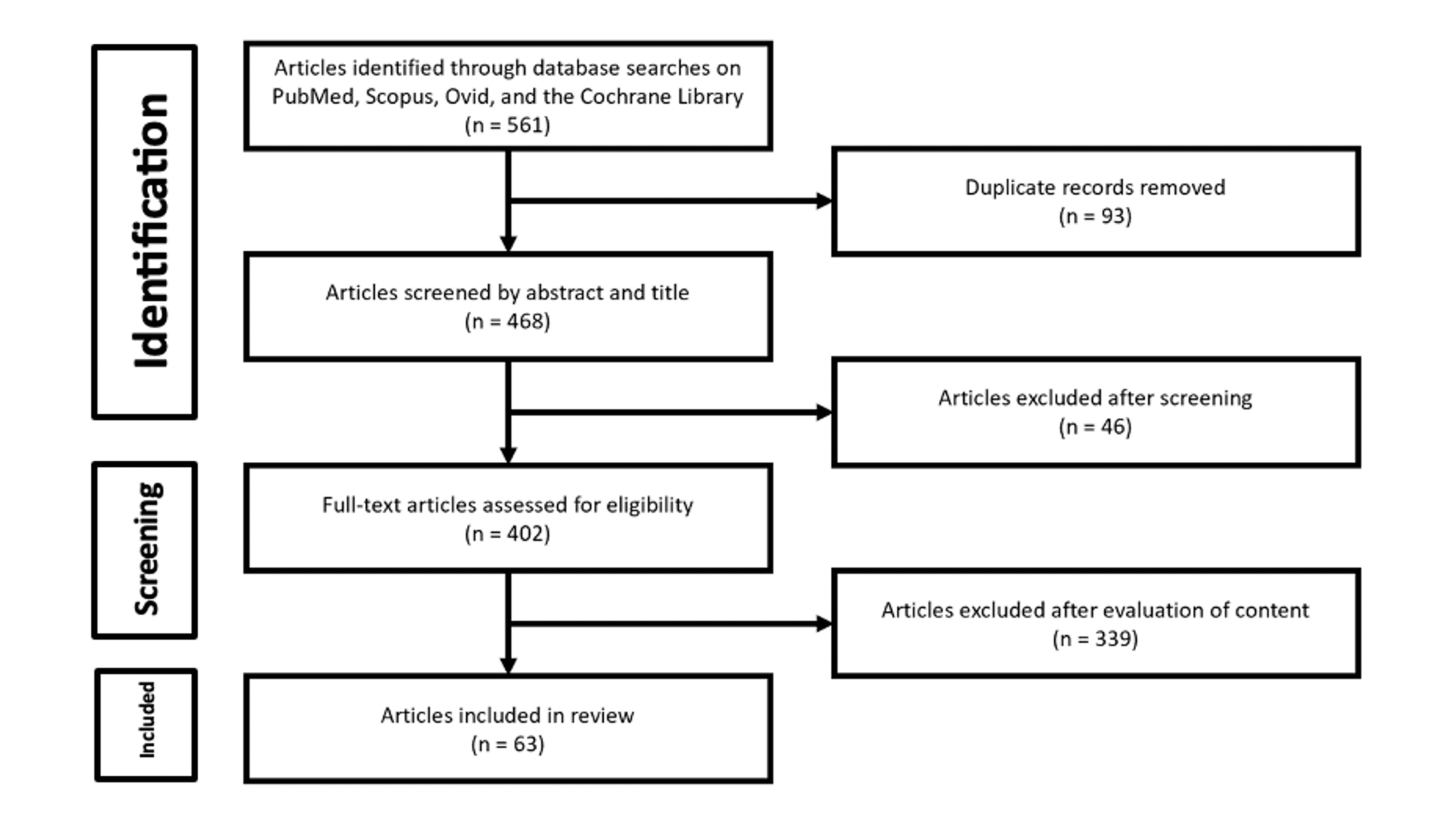
A schematic diagram showing the PRISMA process for screening search results. Narrowed results included 63 articles in the final review. PRISMA: Preferred Reporting Items for Systematic Reviews and Meta-Analyses

A total of 561 articles were retrieved, of which 93 were duplicate results. Of the 468 unique results, 46 were excluded after a title/abstract screening as they did not have a full-text English translation available. The remaining 402 articles were subjected to full-text evaluation and 63 articles met our inclusion criteria after a full-text review (Figure 1).

**Figure 2:**
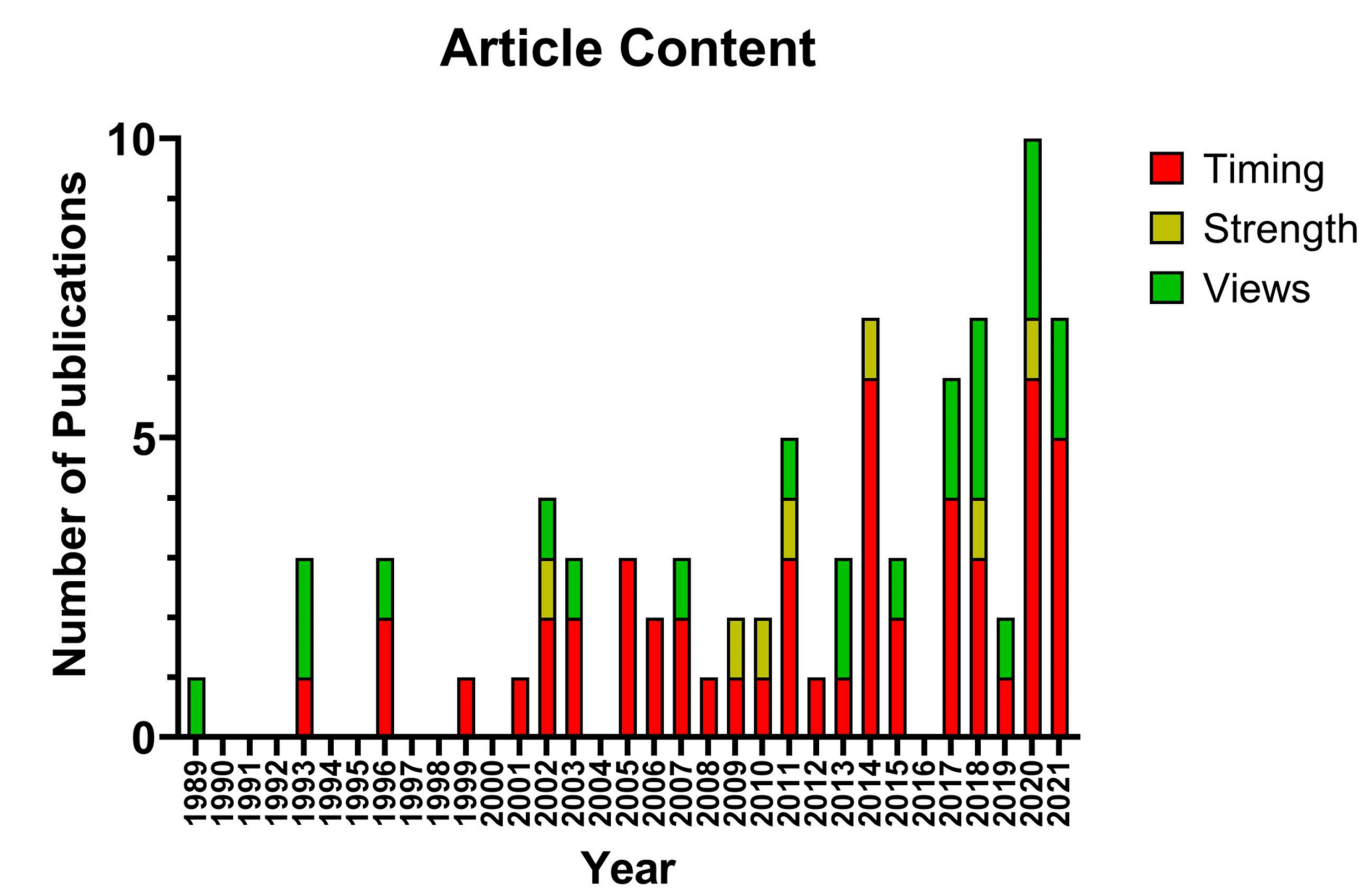
A graph depicting the distribution of included articles based on category of content matter with respect to time.

An analysis of the content of each article revealed that 22 articles focused on specific imaging views, 51 on the timing of the postoperative MRI, and seven on the strength of magnetic fields used (Figure 2).

Several articles dealt with more than one class of variable of interest. The articles were published during the period between January 1, 1989 and March 31, 2022. The majority of articles (41/63, 65.1%) were published after 2010.

We identified several variations of imaging algorithms based on the specific imaging views, timing of postoperative MRI, and strength of magnetic fields used. The articles included in our review were predominantly systematic reviews and cohort studies, so the highest level of evidence rating that we awarded to any specific factor was IA [16,17]. The levels of evidence classifications associated with each identified factor are listed in Table 1.

**Table 1:** A table summarizing the variations of each imaging algorithm component identified in the literature review. For each variation, the level of evidence supporting its use is included.

Algorithm Component	Variations	Level of Evidence
Timing	Intraoperative	IIC
	EPO<24 hours	IIC
	EPO<48 hours	IIC
	EPO<72 hours	Inconclusive
	EPO<1 week	IIA
	EPO <2 weeks	Inconclusive
	EPO < 90 days	Inconclusive
	LPO	IIA
Strength	0.15 Tesla	IIC
	1.5 Tesla	IA
	3.0 Tesla	IIA
Imaging Sequence	NC T1 sequence	IA
	NC T2 sequence	IA
	C+ T1 sequence	IA
	C+ T2 sequence	IIB
	DC+T1 or T2 sequence	IIC
	DWI	IIB
	VIBE	IIB

Discussion

Timing: Intraoperative MRI

A total of 12 studies investigated the efficacy of intraoperative MRI (iMRI) in transsphenoidal adenoma resection [19-30]. Of these publications, eight studies reported that iMRI provided surgeons with immediate identification of residual tumors following endoscopic resection [19-26]. Identifying remnant tumors enabled surgeons to further resect accessible residual tumors during the same procedure to reduce the requirement for later reintervention. Based on these eight studies, iMRI enabled additional tumor resection in 9.6-29.7% of cases [19-26]. However, these same studies reported an incidence of reoperation without additional tumor resection in 11-26.3% of cases [19-26]. There is an increased hospital cost associated with additional surgery, although the cost-benefit comparison of attempting additional resection following iMRI remains unclear. Three of the eight studies found that iMRI significantly increased the rates of progression-free survival in the years following iMRI resection surgery, with Bellut et al. (2010) reporting a 5.1% increase in remission rate following additional resection using iMRI [19-26].

In contrast, four studies investigating the efficacy of iMRI for adenoma resection reported no clear benefit to using iMRI over conventional LPO MRI [27-30]. An early study by Nimsky et al. (2004) using a low-field 0.2 Tesla scanner reported insufficient image quality for reliably estimating the volume of parasellar residual tumors and found that the higher resolution of routine pre- and postoperative imaging with 1.5T machines far outweighed the benefit of 0.2T iMRI [27]. Wu et al. (2009) reached a similar conclusion regarding 0.15T iMRI [28]. Next, Møller et al. (2018) found no significant difference in the GTR rate, mean tumor remnant volume, or tumor resection volume when using iMRI as compared to without using iMRI [29]. Finally, a meta-analysis by Soneru et al. (2019) reported similar rates of GTR with or without iMRI during endoscopic transsphenoidal resection [30]. Given the greater cost of iMRI compared to conventional MRI, these studies favored the use of standard LPO imaging. *Timing: EPO ≤24 Hours, 48 Hours, 72 Hours*

From our review, six studies utilized MRI within 24 hours following transsphenoidal adenoma resection to evaluate the extent of remnant tumors [7,9,31-34]. Of these studies, only one supported the use of 24-hour EPO MRI [9]. Stofko et al. (2014) concluded that 3T MRI immediately following endoscopic endonasal resection of pituitary lesions provided accurate and reliable information regarding the presence of residual tumors compared to reconstruction and packing materials [9].

However, results from five of the six studies supported the use of three, six, or nine-month LPO MRI over 24-hour EPO MRI [7,31-34]. A study correlating the postoperative macroadenoma volume with clinical outcomes showed persistent mass-like abnormalities in 87% of patients on 24-hour EPO MRI [31]. However, imaging at three months showed that only 18% of patients had an overall tumor resection volume of < 75% and only 22% of patients failed to demonstrate significant improvement of symptoms at three, six, and nine months after surgery [31]. This suggests that 24-hour EPO MRI might be inaccurate in detecting remnant tumors and a poor predictor of long-term symptom improvement compared to LPO MRI at three, six, or nine months postoperatively [31]. In concordance with this aforementioned study, four other studies using both 24-hour EPO MRI and three-month LPO MRI for evaluating the extent of GTR favored the use of three-month LPO MRI when reporting results [7,32,33,34]. In particular, Kremer et al. (2002) explicitly stated that 24-hour EPO MRI was ineffective in detecting residual adenoma tissue using 1.5T MRI, noting that interpretation of postoperative images for detection of residual adenoma was much improved using three-month LPO MRI [7]. We identified four studies investigating 48-hour EPO MRI following pituitary adenoma resection [5,35-37]. Of these four studies, two supported the use of 48-hour EPO MRI, one favored LPO MRI at 4-6 months over 48-hour EPO MRI, and one used 48-hour EPO as a reference for iMRI [5,35-37]. Notably, a retrospective cohort study on 102 patients by Alhilali et al. (2020) found that 48-hour EPO MRI had significantly greater sensitivity for detecting residual tumors compared to three-month LPO MRI, with a 100% negative predictive value [5]. Likewise, López et al. (2017) calculated that 48-hour EPO MRI had a sensitivity of 0.78 and a specificity of 0.82 based on their analysis of 73 cases [35]. However, Wang et al. (2021) concluded that LPO MRI 4-6 months after surgery was preferable to 48-hour EPO MRI because it allowed time for hemostatic material to be absorbed and for pituitary tissue to re-expand [36]. We identified two studies investigating the use of EPO MRI at 72 hours following pituitary adenoma resection, one of which used 72-hour EPO as a reference for iMRI [30,38]. Interestingly, one retrospective cohort study of 65 patients determined that the distance between the resection cavity and optic chiasm grew from the 72-hour EPO period to the three-month LPO period [38]. The authors suggested that radiotherapy planning should be deferred to at least three months postoperatively and ultimately supported the use of LPO MRI [38]. The second study concorded in its support of LPO MRI over 72-hour EPO MRI [30]. *Timing: EPO ≤1 Week*

A systematic review of 37 studies by Patel et al. (2018) compared one-week EPO MRI against three-month LPO MRI [10]. The authors concluded that high-resolution one-week EPO MRI can accurately distinguish postoperative changes from residual tumors, but they also concluded that lifetime MRI follow-up remains necessary [10]. They reported that MRI changes should be expected between one week and three months postoperatively [10]. These findings are corroborated by Saeki et al. (2003), who found that the posterior pituitary bright spot descended as the mass effect from the tumor disappeared [39]. A prospective cohort study of 30 patients concluded that one-week EPO MRI with T1 diffusion-weighted imaging (DWI) was more effective in differentiating residual adenoma from postsurgical change compared to contrast-enhanced MRI [2]. A second prospective cohort study of 16 patients further noted that one-week EPO MRI could identify tumor remnants in macroadenomas, but that MRI in general was not useful for postoperative evaluation of microadenomas [39]. *Timing: EPO ≤2 Weeks*

A prospective cohort study of 14 patients by Rajaraman et al. (1999) was the only study looking at EPO MRI within two weeks of surgery [8]. The authors compared two-week EPO to LPO MRI between 3-4 months postsurgery [8]. Because of potential residual tumors, edema, postoperative hemorrhage, and hemostatic material, they found that two-week EPO and preoperative MRI scans were very similar, supporting waiting until at least four months postsurgery in clinically stable patients to allow postsurgical changes to dissipate [8]. *Timing: EPO <90 days*

A study by Kunigelis et al. (2020) was the one to compare MRI <90 days versus ≥90 days postoperatively, which they defined as EPO and LPO, respectively [40]. Their retrospective study of 443 patients found that, in the <90 days category, MRIs resulted in higher rates of re-operation due to some postsurgical changes being mistaken for residual tumors [40]. However, the authors noted that this higher rate of re-operation did not lead to any significant differences in long-term clinical outcomes [40]. As such, they concluded that LPO MRI of ≥90 days is able to minimize costs without sacrificing quality of care [40]. *Timing: LPO ≥90 days*

We identified 24 studies using ≥90-day LPO MRI as a control or supporting ≥90-day LPO MRI over EPO MRI following pituitary adenoma resection [5,7-10,19-38]. There were 12 iMRI studies, six 24-hour EPO studies, four 48-hour EPO studies, two 72-hour EPO studies, one 1-week EPO, and one two-week EPO study that evaluated the efficacy of their respective EPO MRI timings against LPO MRI [5,7-10,19-38]. The vast majority of studies supported the use of ≥90-day LPO MRI, keeping in line with the current standard of care. They all converged on a similar justification: identifying residual adenoma on EPO MRI is less effective than LPO MRI because of acute postoperative changes such as hemorrhagic accumulation or cavity enlargement. Over a ≥90-day course of patient recovery, acute postoperative changes become less apparent as the hemostatic material is absorbed and pituitary tissue re-expands. Thus, LPO MRI becomes more interpretable and more accurate in determining the presence of residual adenoma. Dissenting studies argued for the use of EPO MRI in conjunction with LPO MRI, but not for the outright replacement of LPO MRI.9,10 Accordingly, the literature supports the current consensus recommendation that the first radiologic study to evaluate the extent of pituitary resection be performed 3-4 months after surgery [6]. *Imaging Technique: T1WI*

T1WI is the current gold standard for MRI of the pituitary gland, and all studies that we identified used T1WI as part of their protocols [41]. The crucial advantage of T1WI is its enhancement of fatty tissue signals and suppression of water signals, which causes the neurohypophysis to appear hyperintense and the optic chiasm and adenohypophysis to be isointense relative to gray matter [13,42]. Ultimately, T1WI is highly effective at visualizing critical structures of the pituitary and surrounding sellar region. I*maging Technique: T2WI*

T2WI is typically used in conjunction with T1WI. The main advantage of T2WI is the enhancement of water signal relative to fatty tissue, allowing for visualization of the optic apparatus more clearly. Additionally, this modality permits the ability to distinguish between pituitary microadenoma subtypes, as growth hormone and ACTH-secreting adenomas are hypointense while microprolactinomas and TSH-secreting microadenomas are hyperintense [14,15,42]. However, T2WI tends to show normal pituitary structures as isointense relative to gray matter [13]. This aspect may reduce the utility of T2WI, although it remains equally as ubiquitous as T1WI in this setting. *Imaging Technique: DWI*

A prospective cohort study by Hassan et al. (2018) investigated the efficacy of DWI in identifying residual tumors and distinguishing them from postsurgical changes [2]. They noted that DWI is effective in this regard, as tumor tissue often has a higher apparent diffusion coefficient (ADC) than postsurgical damaged cells [2]. This can be difficult to interpret due to distortion of the signal skull base given the air, bone, and tissue interface. ADC is a measure of the impedance of a tissue to random water molecule diffusion, which is the method by which DWI generates an image [2]. As one of the main concerns of EPO MRI is confusion of benign postsurgical change for residual tumors, DWI provides a potential solution to the challenges created by the timing of EPO MRIs. *Imaging Technique: Volumetric Interpreted Breath Hold (VIBE)*

A prospective cohort study by Davis et al. (2013) found that VIBE was superior to coronal T1WI at visualizing the pituitary gland and cavernous sinus [43]. This technique was unusual for brain imaging as it is generally used for abdominal MRI to decrease patient movement while imaging [43]. However, because this study was the only one that we identified investigating VIBE imaging of the pituitary, it is difficult to properly evaluate the merits of this technique. *Selection of Contrast Agents*

The specific choice of contrast agent may influence the information obtained during postoperative imaging. Several of the studies in our review used gadolinium-based contrasts to shorten the T1 or T2 relaxation time, leading to higher signal intensity on T1WI and lower signal intensity on T2WI. Notably, Parizel et al. (1989) used gadolinium 1,4,7,10-tetraazacyclododecane-N,N’,N’,N”-tetraacetic acid (Gd-DOTA) in a prospective cohort study and found that, compared to NC T1WI and T2WI, the use of Gd-DOTA in T1WI helped to better define the anatomy of tumors and differentiate between tumors and edema [44]. The authors concluded that Gd-DOTA improved the sensitivity and specificity of postoperative residual tumor detection. Another retrospective cohort study by Chakrabortty et al. (1993) used gadolinium-diethylenetriaminepenta-acetic acid (Gd-DTPA) and found that Gd-DTPA allowed for greater enhancement of the host pituitary relative to the adenoma in T1WI compared to NC T1WI [45]. Interestingly, Vitaz et al. (2011) used an unspecified contrast agent in a prospective cohort study of iMRI and noted that the use of this agent in iMRI provided valuable and immediate feedback as to the extent of tumor resection [22]. *Without Contrast*

One study in our review of the literature critiqued the inclusion of contrast administration altogether. A study by Gohla et al. (2020) found that C+ T1WI and NC T2WI have similar specificity and sensitivity [21]. The authors thus questioned the use of gadolinium-based contrast, which carries increased risks of allergic reaction, exacerbation of severe kidney disease, and perioperative complications as well as increased cost [21]. However, there is substantial data that demonstrates the benefits of gadolinium-based contrast in terms of improved residual tumor detection [21]. *Dynamic Contrast*

The use of dynamic contrast must also be considered when comparing pituitary MRI techniques [35,46]. This technique involves injecting a paramagnetic contrast agent into the vascular system multiple times throughout the imaging, so that the signal during and after arrival of the contrast agent can be measured [46]. This contrast agent alters the MRI signal intensity of the surrounding tissue depending on its specific concentration [46]. A retrospective cohort study by López et al. (2017) found that dynamic contrast as part of an MRI protocol could reliably assess the degree of pituitary tumor resection [35]. *Magnetic Field Strength*

We identified three studies that evaluated the efficacy of 0.15T MRI in identifying residual adenomas [24,29,30]. Two of those studies reported no significant benefit to 0.15T MRI over standard 1.5T MRI imaging [29,30]. Specifically, these studies concluded that 0.15T MRI is not sufficient in estimating the amount of parasellar residual tumors given the false or uncertain images generated by low-field MRI because of difficulty in discriminating between remnant tumors and blood within the venous sinus [29,30]. However, a third study reported 0.15T MRI to be safe, highly effective, and increase remission rates [20]. Given that all 3 studies utilized 0.15T MRI as part of an iMRI protocol, the true value of such a low field strength in the EPO and LPO time period is likely limited. *1.5 Tesla MRI*

We identified 13 studies using 1.5T MRI at various postoperative timepoints and fields of view [2,8-10,21,23,26,30,31,37,41,47-49]. Studies using 1.5T EPO MRI showed mixed results. Some studies reported that 1.5T MRI in the EPO period provides accurate and reliable information regarding the presence of residual tumors compared to reconstruction and packing materials [9]. In contrast, other studies concluded that the appearance of the sellar contents on 1.5T EPO MRI appear remarkably similar to that seen before surgery, even after technically adequate resection, due to a combination of residual tumors, edema, postoperative hemorrhage, and hemostatic material that may be present [8]. Despite the mixed findings of these EPO MRI studies, all LPO studies that we encountered used 1.5T MRI, thus showing consistent support for the reliability of 1.5T LPO MRI in identifying residual adenoma [2,8-10,21,23,26,30,31,37,41,47-49]. *3.0 Tesla MRI*

We identified six studies using 3T MRI at different timepoints and fields of view [9,10,21,36,50,51]. Similar to 1.5T MRI, we found that studies using 3T MRI in the EPO period showed mixed results while 3T MRI studies in the LPO period all supported ≥90-day LPO imaging to be reliable in detecting residual tumors [9,10,21,36,50,51]. These studies noted improved resorption of hemostatic material, better discrimination of the packing material from endogenous tissue, and postoperative pruning of the pituitary gland closer to its preoperative size during the LPO period over the EPO period [36,50].

Discussion on Timing

Our review of the literature revealed Level IIA evidence in support of LPO MRI performed three months or more. The evidence regarding other EPO timepoints was not as compelling or homogeneous as for EPO MRI at one week. We found Level IIC evidence in favor of routine utilization of EPO MRI within 24 hours and within 48 hours, but the current literature still demonstrated the superiority of LPO MRI in both cases. Intriguingly, we found no evidence to support the use of EPO MRI within 72 hours, within two weeks, or within 90 days. It is also worth noting that we found Level IIC evidence in favor of routine utilization of iMRI, but the increased cost and logistical difficulty in implementation reduce its viability as a practical alternative or adjunct.

Thus, the strength of evidence supporting EPO MRI at one week appears somewhat unusual and requires further investigation. Current guidelines support LPO MRI of at least three months postoperation, but the downside of this is that the presence of residual tumors cannot be determined immediately, something which influences postoperative treatment. Only 50-80% of pituitary tumor resections result in gross total resection, meaning that a significant number of operations involve residual tumors [3]. With EPO, earlier detection of residual tumors can be achieved, and subsequent MRI scans can still be able to detect tumor recurrence. A downside of EPO is that it may detect immediate postoperative benign complications such as asymptomatic hematoma and cause unnecessary stress to the patient. Previous studies have found the incidence of asymptomatic hematoma to be as high as 16.5%.12 Our recommendation, therefore, is in agreement with the current guideline, which is to perform LPO MRI at three months. *Discussion on Imaging*

Our review of the literature revealed Level IA evidence in support of T1WI and T2WI as part of the pituitary imaging algorithm. As pituitary microadenomas can range from hypointense to hyperintense on T1WI and T2WI depending on the adenoma subtype, a combination of T1WI and T2WI is necessary to attain clear visualization of the diaphragma sellae and surrounding structures. As we found only Level IIB evidence in support of DWI and VIBE, we do not recommend the use of these techniques at this time, although future studies may elucidate certain advantages of these alternative methods. Our recommendation, simply, is to adhere to the current gold standard of obtaining T1WI and T2WI.

Furthermore, our literature review noted Level IA evidence in support of the coronal and sagittal views. However, we noted only Level IIC evidence in support of the axial views. Accordingly, our recommendation is to exclude axial views and preferentially use coronal and sagittal views of the pituitary gland and surrounding structures.

To continue, our literature review found Level IA evidence for the use of NC T1WI, NC T2WI, and C+ T1WI and Level IIB evidence for the use of C+ T2WI. As there is Level IIC evidence in favor of any given gadolinium-based contrast agent, we do not have a recommendation for any specific compound. It is worth mentioning that the Level IIC evidence supporting the use of DC+ imaging is promising; however, there is insufficient evidence at the time of our study to formally include it in a routine imaging protocol. Thus, our recommendation is to use C+ T1WI and NC T2WI, given its particular advantage in the postoperative setting, although further research may prove the utility of including DC+ T1WI and/or C+ T2WI. As per standard procedure, we advise against the use of contrast in any patients with known allergies to the contrast agent or pre-existing severe kidney disease. *Discussion on Magnetic Field Strength*

We found 1.5T MRI has Level IA evidence to support its use while 3T MRI has Level IIA evidence. While studies investigating 1.5T MRI in the EPO period showed mixed results, all studies evaluating 1.5T MRI in the LPO period supported the efficacy of 1.5T MRI. In contrast, 0.15T MRI had considerably less support, with two of the three 0.15T MRI studies reporting no significant benefit to 0.15T MRI over standard LPO imaging. Finally, 3T MRI showed similar findings as 1.5T MRI. Studies investigating 3T MRI in the EPO period had mixed results, while 3T MRI in the LPO period was consistently considered reliable for distinguishing residual adenoma. Because both 1.5T and 3T MRI literature showed similar trends of mixed results for EPO but consistent support for LPO, we conclude that timing is the limiting factor for reliably distinguishing residual adenoma rather than MRI magnetic strength. One factor that likely influenced the greater preponderance of literature for 1.5T compared to 3T is the increased cost of 3T MRI machines. However, as technology progresses and costs decrease, it is likely that we see a greater emphasis on 3T MRI relative to lesser magnetic strengths moving forward. *Proposed Algorithm*

Our group recommends the routine use of coronal and sagittal NC T1WI, NC T2WI, and C+ T1WI using 1.5T MRI at three months postsurgical resection of pituitary tumors based on the highest levels of evidence available for each of these aspects of our proposed imaging algorithm.

## Conclusions

Our systematic review of early EPO MRI for transsphenoidal pituitary surgery reveals advancements in imaging protocols that could impact pituitary adenoma management. Although LPO MRI, typically at 3-4 months after surgery, remains the standard, new imaging options like iMRI and high-field strength MRI (3T) suggest potential advantages of earlier imaging. Traditional concerns about EPO MRI focus on difficulties interpreting early scans due to postoperative changes, such as hemorrhage and edema, which may obscure residual tumor tissue. However, newer imaging techniques, like dynamic contrast-enhanced MRI and DWI, have shown promise in detecting residual tumors earlier than previously feasible.

Despite these technological improvements, evidence comparing EPO and LPO MRI outcomes remains mixed. Our review indicates that while EPO MRI can identify residual tumors within 24-72 hours post-surgery, it may not reliably predict long-term outcomes as effectively as LPO MRI. Additionally, the cost and risk of false positives with EPO MRI may limit its practicality in some clinical settings. iMRI during surgery has demonstrated value by improving the rate of gross total resection, which could reduce the need for repeat surgeries. Moving forward, a tailored approach using both EPO and LPO MRI might be ideal, integrating early assessments for high-risk patients while maintaining LPO MRI as the gold standard for long-term evaluation.
